# Bone metastases do not affect the measurement uncertainties of a global digital volume correlation algorithm

**DOI:** 10.3389/fbioe.2023.1152358

**Published:** 2023-03-16

**Authors:** Giulia Cavazzoni, Luca Cristofolini, Enrico Dall’Ara, Marco Palanca

**Affiliations:** ^1^ Department of Industrial Engineering, School of Engineering and Architecture, Alma Mater Studiorum-University of Bologna, Bologna, Italy; ^2^ Department of Oncology and Metabolism, The University of Sheffield, Sheffield, United Kingdom; ^3^ INSIGNEO Institute for in Silico Medicine, The University of Sheffield, Sheffield, United Kingdom

**Keywords:** spine metastases, human vertebrae, digital volume correlation (DVC), measurement uncertainty, microstructure

## Abstract

**Introduction:** Measurement uncertainties of Digital Volume Correlation (DVC) are influenced by several factors, like input images quality, correlation algorithm, bone type, etc. However, it is still unknown if highly heterogeneous trabecular microstructures, typical of lytic and blastic metastases, affect the precision of DVC measurements.

**Methods:** Fifteen metastatic and nine healthy vertebral bodies were scanned twice in zero-strain conditions with a micro-computed tomography (isotropic voxel size = 39 μm). The bone microstructural parameters (Bone Volume Fraction, Structure Thickness, Structure Separation, Structure Number) were calculated. Displacements and strains were evaluated through a global DVC approach (BoneDVC). The relationship between the standard deviation of the error (SDER) and the microstructural parameters was investigated in the entire vertebrae. To evaluate to what extent the measurement uncertainty is influenced by the microstructure, similar relationships were assessed within sub-regions of interest.

**Results:** Higher variability in the SDER was found for metastatic vertebrae compared to the healthy ones (range 91-1030 με versus 222–599 με). A weak correlation was found between the SDER and the Structure Separation in metastatic vertebrae and in the sub-regions of interest, highlighting that the heterogenous trabecular microstructure only weakly affects the measurement uncertainties of BoneDVC. No correlation was found for the other microstructural parameters. The spatial distribution of the strain measurement uncertainties seemed to be associated with regions with reduced greyscale gradient variation in the microCT images.

**Discussion:** Measurement uncertainties cannot be taken for granted but need to be assessed in each single application of the DVC to consider the minimum unavoidable measurement uncertainty when interpreting the results.

## 1 Introduction

**FIGURE 1:**
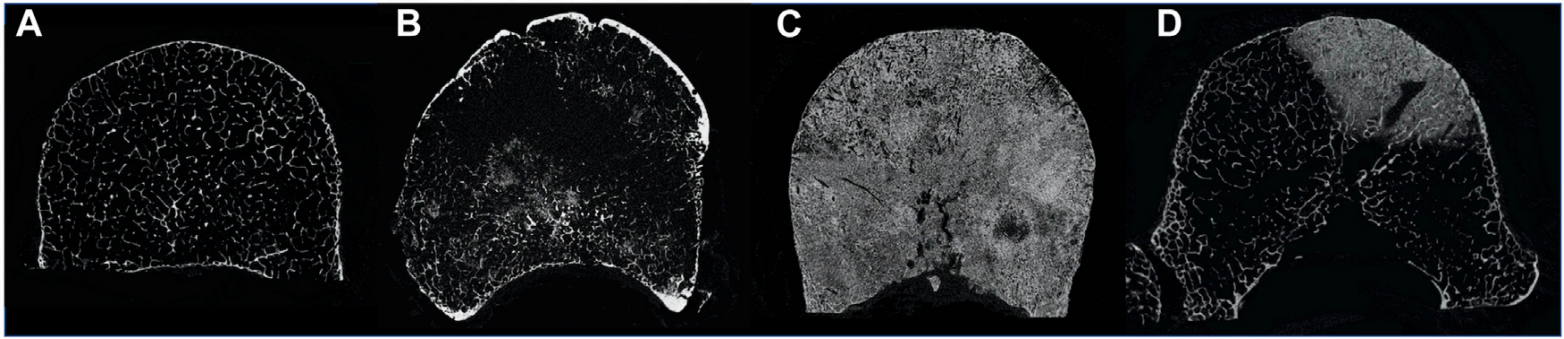
MicroCT cross section of a healthy vertebra **(A)**, a vertebra with lytic metastases **(B)**, a vertebra with blastic metastases **(C)** and a vertebra with mixed metastases **(D)**.

Bone is the most common site affected by metastatic disease (Coleman, 1994). The axial skeleton, and in particular the spine, is the anatomical site where most commonly metastatic lesions form (Coleman, 1994, 2001). Bone metastases are malignant formations that alter the physiologic bone cells activity leading to an unbalanced homeostasis (Selvaggi and Scagliotti, 2005; Whyne, 2014). As a consequence, the internal microstructure of the vertebrae is altered (Wise-Milestone et al., 2012; Burke et al., 2017), resulting in reduced (i.e., lytic metastases) and/or increased (i.e., blastic metastases) bone mineral density. This compromises the internal microstructural optimization (Figure 1) (Burke et al., 2017) and this, in general, is associated with a reduced ability to withstand physiological load (Nazarian et al., 2006; Perilli et al., 2012). In particular, in case of metastatic vertebrae, the higher is the metastatic involvement the higher is the risk of fracture (Whyne, 2014). Thus, investigation of the biomechanics of metastatic vertebrae in elastic regime and at failure is important to quantify their mechanical properties, in order to assess the spine stability and define better predictors of the risk of fracture.

Local internal experimental strain measurements of whole bones can be performed by using Digital Volume Correlation (DVC) (Dall’Ara and Tozzi, 2022), processing micro-Computed Tomography (microCT) images of the object in unloaded and loaded conditions. This contact-less technique provides a volumetric measurement of the displacement field and, by differentiation, of the strain fields within the imaged structure. DVC has already been applied to evaluate the internal displacement and strain fields in different musculoskeletal tissues (Dall’Ara and Tozzi, 2022; Dall’Ara et al., 2022). In particular, this approach has already been used to study the biomechanics of animal (Danesi et al., 2016; Palanca et al., 2016; Tozzi et al., 2016; Palanca et al., 2021b) and human (Hussein et al., 2012) vertebrae and to investigate the local strain distributions within the whole vertebral body and in specific subregions. However, DVC accuracy and precision may be affected by the bone structure (Liu and Morgan, 2007). Moreover, as there is not another precise method to measure the internal displacement and strain fields, the assessment of the measurement uncertainties of the DVC is usually performed with “zero-strain” test (Liu and Morgan, 2007; Dall’Ara et al., 2014), which consists in processing with the DVC algorithm two repeated scans of the same specimen in the unloaded configuration (“zero-strain” condition). In fact, while in this case the loading condition is simplified, it allows to quantify the effect of some sources of errors (e.g., image noise and image processing parameters). Wide ranges of measurement uncertainties have been reported in the literature for different DVC approaches, spatial resolution and bone types (Roberts et al., 2014). In particular, displacement and strain measurement uncertainties of the DVC were found to decrease following a power law when the measurement spatial resolution increase (i.e., the larger the measurement spatial resolution the lower the measurement uncertainty) (Dall’Ara et al., 2014; Palanca et al., 2015; Dall’Ara et al., 2017). Source of errors include the quality of the input microCT images (signal to noise ratio, SNR) (Dall’Ara et al., 2017), the used correlation algorithm, and the individual operational parameters within the DVC algorithm (Roberts et al., 2014; Palanca et al., 2015). Nevertheless, the bone microstructure may also play a fundamental role in assessing the displacement and strain fields with the DVC. Liu and Morgan (Liu and Morgan, 2007) found that DVC uncertainties were affected by different bone microstructures scanned with microCT, including bovine and rabbit distal femur, bovine and rabbit proximal tibia, rabbit and human vertebral body (precision ranged 345–794 με across all bone types). In order to better understand how the vertebral microstructure could explain differences in performance of DVC approaches, DVC displacement and strain measurement uncertainties were also investigated for porcine vertebrae with cement augmentation (Tozzi et al., 2017) and with induced bone lesions replicating the phenotype of lytic metastases (Palanca et al., 2021b). However, it is still unknown how the DVC measurement uncertainties are affected by actual metastatic lesions in human vertebrae.

The aim of the study was to understand if there is a relationship between the microstructural parameters of vertebrae with/without metastatic lesions and the measurement uncertainties of a global DVC approach. In particular, this study investigated: 1) the relationship between the displacement measurement uncertainties and the measurement spatial resolution; 2) the relationship between the strain measurement uncertainties and the measurement spatial resolution; 3) the relationship between the strain measurement uncertainties and the microstructural parameters in the whole vertebra (metastatic: highly heterogeneous and healthy: less heterogeneous), and in sub-regions of interest.

## 2 Materials and methods

### 2.1 Sample and imaging

The study was approved by both the Bioethics Committee of the University of Bologna (reference n. 17325, 8th February 2019) and The University of Sheffield (reference n. 031782, 22nd June 2020). The work was performed in accordance with the Declaration of Helsinki. Eleven spines from donors (5 males and 6 females, 68 ± 13 years old, Tables 1, 2) with medical history of spinal metastases, previously used in (Palanca et al., 2021a), were obtained from an ethically approved donation program (Anatomy Gift Registry, AGR).

**TABLE 1:** Main microstructural parameters calculated for each metastatic vertebra.

Specimen ID	Donor	Age	Sex	Group	Spine level	BV/TV Mean [%]	St.Th.	St.Sp.	St.N.
							Mean ± SD [μm]	Mean ± SD [μm]	Mean [1/mm]
1	A	81	M	Lytic	L1	11.3	166 ± 79	841 ± 339	0.68
2	B	59	F	lytic	T8	9.6	182 ± 109	1,022 ± 337	0.53
3	B	59	F	lytic	T11	8.4	161 ± 88	1,004 ± 353	0.52
4	C	82	F	lytic	T11	11.6	155 ± 70	856 ± 406	0.75
5	D	46	F	lytic	T12	7.5	175 ± 101	1,279 ± 849	0.43
6	E	72	M	lytic	T6	14.1	191 ± 94	1,036 ± 706	0.74
7	F	66	M	blastic	L2	72.6	198 ± 80	1,298 ± 75	3.66
8	G	78	M	blastic	L1	27.7	398 ± 223	880 ± 400	0.69
9	G	78	M	blastic	L2	45.4	250 ± 121	372 ± 231	1.81
10	G	78	M	blastic	L4	54.4	306 ± 174	356 ± 339	1.78
11	G	78	M	blastic	L5	23.3	189 ± 108	600 ± 419	1.23
12	H	55	F	mixed	T11	19.6	251 ± 142	1,030 ± 660	0.78
13	I	83	M	mixed	L4	52.2	328 ± 163	610 ± 421	1.59
14	J	73	F	mixed	T12	16.6	211 ± 107	1,029 ± 491	0.79
15	J	73	F	mixed	L4	51.0	276 ± 122	360 ± 227	1.85
Mean ± SD		73 ± 11	—	—	—	28.4 ± 21	229 ± 71	838 ± 313	1.19 ± 0.85
Range		46–83	—	—	—	7.5–72.6	155–398	356–1,298	0.43–3.66

**TABLE 2:** Main microstructural parameters calculated for each control vertebra.

Specimen ID	Donor	Age	Sex	Group	Spine level	BV/TV Mean [%]	St.Th.	St.Sp.	St.N.
							Mean ± SD [μm]	Mean ± SD [μm]	Mean [1/mm]
16	B	59	F	Control	T7	7.4	160 ± 85	1,078 ± 369	0.46
17	B	59	F	control	T10	8.0	156 ± 78	1,039 ± 366	0.05
18	K	51	F	control	L3	8.0	157 ± 66	1,182 ± 368	0.51
19	K	51	F	control	T4	8.6	159 ± 74	1,094 ± 340	0.54
20	C	82	F	control	T10	12.3	154 ± 67	782 ± 242	0.80
21	D	46	F	control	T5	10.1	163 ± 84	961 ± 593	0.62
22	J	73	F	control	T11	9.3	158 ± 70	1,037 ± 425	0.59
23	J	73	F	control	L3	9.1	177 ± 101	1,126 ± 464	0.52
24	E	72	M	control	T7	14.5	167 ± 63	830 ± 298	0.87
Mean ± SD		63 ± 13	—	—	—	9.7 ± 2.3	161 ± 7	1,014 ± 134	0.55 ± 0.23
Range		46–82	-	—	—	7.4–12.3	156–177	782–1,182	0.05–0.80

Clinical CT scans (voxel size of 0.45 × 0.45 × 1 mm^3^ (Palanca et al., 2021a)) of the spines were used to identify vertebrae with metastases and healthy vertebrae (i.e., without any radiological sign of metastatic lesions, later referred to as “control”). Fifteen metastatic vertebrae (Table 1) and nine control vertebrae (Table 2) were selected for this study.

In particular, vertebrae with lytic (6), blastic (5) and mixed (4) metastases were included to enlarge the ranges of the microstructural parameters. Vertebrae from the thoracolumbar spine (T4 to L5) were dissected and the posterior elements were removed.

Each vertebral body was scanned with a microCT (VivaCT80, Scanco Medical, Switzerland) within a radiotransparent custom-built loading jig (Ryan et al., 2020; Palanca et al., 2021b) equipped with a uniaxial load cell (10 kN, HBM, Germany). Each specimen was scanned so that the anterior side of the vertebral body was on the superior side of the microCT cross section (the cranial-caudal axis of the vertebra was roughly aligned to the microCT longitudinal axis). The following scanning parameters were used (Costa, 2020; Palanca et al., 2021b): current 114 μA, voltage 70 kVp, integration time 300 ms, power 8 W, 750 projections/180°, isotropic voxel size of 39 μm. These parameters enabled the scan of the whole vertebral body in a reasonable time (∼1–1.5 h), which was a requirement of the study for future time-lapsed mechanical testing and characterization of the biomechanical properties of the bone under different load levels. The standard reconstruction algorithm recommended by the manufacturer was used and to reduce the beam hardening artefacts a polynomial correction based on scans of a wedge phantom with 1,200 mg/cm^3^ of hydroxyapatite (HA) was used (Kazakia et al., 2008). Each specimen was thawed 24 h in a fridge (4°C) and 1 hour at room temperature (21°C) before the test, then it was wrapped in gauzes soaked in saline solution. A pre-load of 50 N was applied to ensure the stability of the specimen inside the jig. Then, each vertebra was scanned twice (Scan1 and Scan2, respectively), with repositioning of the jig inside the microCT chamber between the scans.

### 2.2 Microstructural properties of metastatic and control vertebrae

**FIGURE 2:**
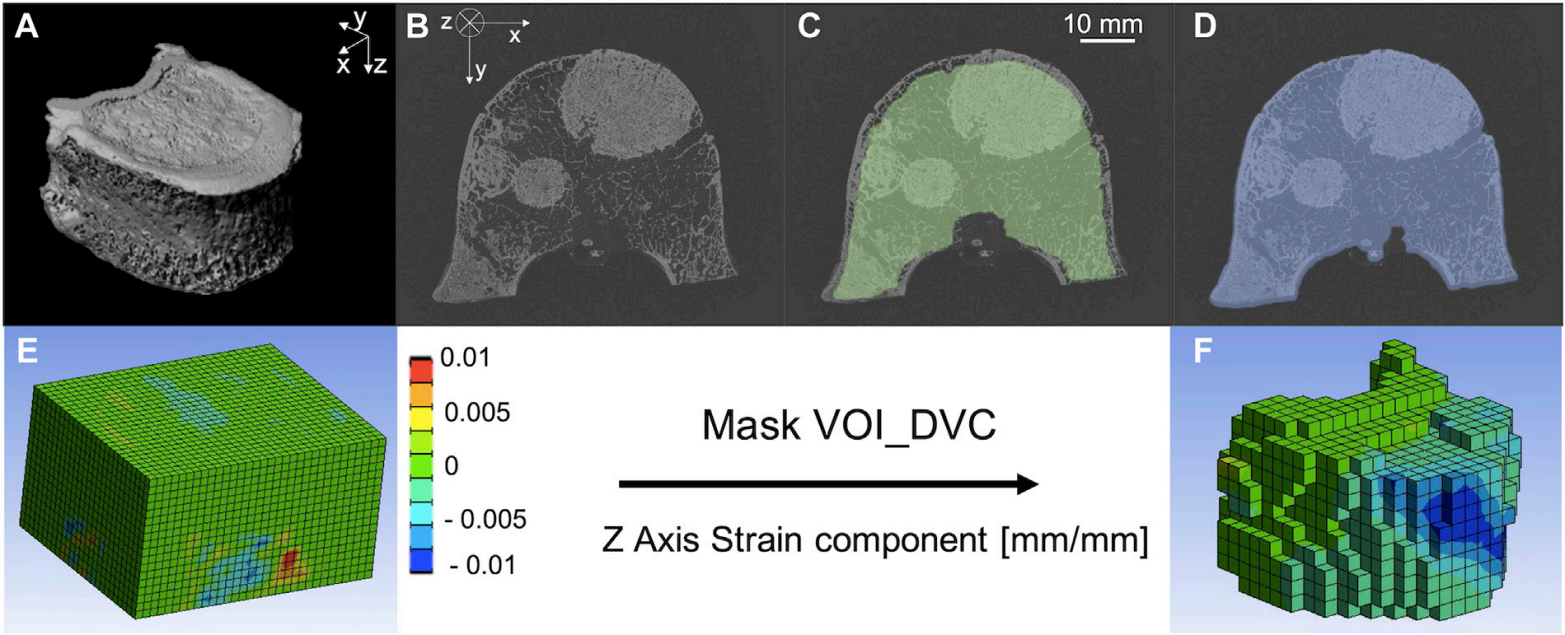
**(A)** microCT of a metastatic vertebra, **(B)** microCT cross section of a metastatic vertebra, **(C)** microCT cross section of a metastatic vertebra with superimposed VOI_NoCort mask in green, **(D)** microCT cross section of a metastatic vertebra with superimposed VOI_DVC mask in blue. Normal component of strain along the cranio-caudal direction (i.e., z—direction) before **(E)** and after **(F)** the application of the Mask VOI_DVC.

Scan1 was used to assess the microstructural trabecular properties of the metastatic and control vertebrae (Nägele et al., 2004; Sone et al., 2004; Tamada et al., 2005; Bouxsein et al., 2010). Air bubbles within the vertebral body (identified as regions with grey scale values close to zero) were virtually removed by using a custom-made script (ImageJ, National Institutes of Health, United States) that replaces these low grey scale values with values similar to those measured for the bone marrow. In order to compute the microstructural parameters, the images were then binarized as follow. A 3D median filter (isotropic support equal to 0.5) was applied to reduce the high frequency noise of the microCT images without reducing the contrast between bone and marrow (Stauber and Müller, 2008; Bouxsein et al., 2010). A single level threshold, calculated as the value identified by the Otsu Thresholding algorithm (ImageJ, National Institute of Health, United States) increased by 5%, was applied to segment the images. This threshold value was determined from a preliminary analysis where corrections of ±5% or ±10% of the automatically calculated Otsu Threshold value were explored. A correction of +5% was found to be the optimal threshold value that best preserved the trabecular structure after visual inspection. In order to perform the microstructural analyses only on the trabecular bone inside the vertebral body, a volume of interest (VOI_NoCort) was defined through a manual segmentation (Amira 6.2, Thermo Fisher Scientific) of the microCT images (Figures 2A, 2B) as the volume of the vertebral body excluding the cortical shell (Figure 2C): the area of the cross section of the vertebral body was manually defined every 20 slices and interpolated using a trilinear interpolation on Amira. Afterwards, the main trabecular 3D microstructural parameters for each metastatic and control vertebra were computed (Danielsson, 1980; Hildebrand and Rüegsegger, 1997; Remy and Thiel, 2002; Bouxsein et al., 2010) in CTAnalyzer (V1.17.7.2, Bruker, MA, United States), (Tables 1, 2).

• Bone volume fraction, (BV/TV, (%)), is calculated as the ratio between the number of the bone voxels (after thresholding) and the total number of voxels included in the VOI_NoCort.
• Trabecular or Structure Thickness (St.Th. (μm)), represents the mean thickness of the trabeculae or other bone structures (e.g., cortical shell and endplates). St.Th. Is calculated using a 3D sphere-fitting method after skeletonization of the structure (trabeculae or bony structure).
• Trabecular or Structure Separation [St.Sp. (μm)], is the mean distance between trabeculae or other bone structures (e.g., cortical shell and endplates). St.Sp. Is calculated using a 3D sphere-fitting method, where the spheres are fitted to the background.
• Trabecular or Structure Number [St.N. (1/mm)] is a linear density measurement assessed as the average number of trabeculae or structure per unit length. St.N. is calculated as the mean distance between the mid-axis of the trabecular or structure.

With the term “Structure” we refer to the mineralised structures within the metastatic vertebral bodies, which may include regions with blastic tissue.

### 2.3 Digital volume correlation

The measurement uncertainties of a global DVC approach [BoneDVC (Barber and Hose, 2005; Dall’Ara et al., 2014)] for metastatic and control vertebrae were evaluated processing the repeated scans (Dall’Ara et al., 2014). Scan1 and Scan2 were prepared as follow: air bubbles were removed from both scans as described in 2.2. This step was particularly important for the DVC analyses as the bubbles may move between the two scans, affecting DVC measurements. Rigid registration between Scan2 and Scan1 (performed in Amira: alignment of principal axes; Lanczos interpolation) was applied to remove any remaining rigid body motions resulting from the repositioning of the specimen inside the microCT chamber. Binary masks (value of 1 for voxels within the vertebral body, values of 0 outside) were created for each image using a Gaussian 3D filter (variance equal to 5, isotropic support equal to 7 voxels), followed by a manually selected single-level threshold value, and a filling algorithm (ImageJ, National Institutes of Health, United States). The binary images for Scan1 and Scan2 were merged and used to restrict the DVC analysis only within the mask.

The DVC operating principles are reported in details elsewhere (Dall’Ara et al., 2014; Palanca et al., 2017). Briefly, Scan1 and Scan2 of each vertebra were elastically registered (Sheffield Image Registration Toolkit, ShIRT) (Barber and Hose, 2005; Barber et al., 2007) to compute the displacement field. The registration algorithm consists in superimposing on both images a regular parallelepiped grid with cubic cells with side length equal to the nodal spacing (NS). ShIRT solves the registration equations at the nodes of the grid within the binary mask maximising the mutual information. The registration equations include the displacement terms and a term to account for potential changes in the grey levels. Trilinear interpolation for displacements is assumed within the cells of the grid. The registration is solved by adding a smoothing coefficient in the displacement field to overcome the poorly conditioned mathematical problem. The problem is then solved iteratively to compensate for potentially large displacements. Considering that for every DVC approach a compromise between the DVC spatial resolution and its accuracy should be accepted (Dall’Ara et al., 2014), the accuracy of the approach for different NSs, spanning between ∼1 mm and ∼4 mm, were investigated (Section 2.4). The strain field is obtained differentiating the displacement field with an FE software package (Mechanical APDL v19, ANSYS, United States). To do so, the hexahedral DVC grid was converted into an 8-nodes hexahedral mesh that was imported in the FE software. The displacement calculated from the elastic registration in each node of the grid was imposed at the nodes of the FE elements and then differentiated into strains. The FE software package was then used to visualize the results in the volume of interest, defined as the volume of the binary mask of the Scan1 reduced by 25 voxels (0.975 mm) in each direction (VOI_DVC) (Figure 2D). All cells of the DVC grid without any node within the VOI_DVC were removed (Voxel detection (Giorgi and Dall’Ara, 2018), Figures 2E, F). This approach was used to exclude boundary regions from the analyses, where the DVC typically performs worse (Palanca et al., 2016).

### 2.4 Metrics to assess the DVC uncertainties

Given the repositioning of the specimen and the zero-strain condition, any variation of the displacement and any strain value different from zero can be considered as an error. The precision of the displacement measurement was calculated for each vertebra as the standard deviation of the measurements (random error) across the nodes of the DVC grid for the three Cartesian components of the displacement: along the left-right (x), anterior-posterior (y), and cranio-caudal (z) direction of the vertebra.

The strain measurement uncertainty was evaluated as the standard deviation (SDER) of the average of the absolute values of the six strain components across the nodes of the DVC grid (Liu and Morgan, 2007; Palanca et al., 2016):

$$SDER=\sqrt{{\frac{1}{N}\overset{N}{\underset{k=1}{\sum }}\left(\frac{1}{6}\overset{6}{\underset{c=1}{\sum }}\left|\varepsilon_{c,k}\right|\right.-MAER)}^{2}}$$

$$MAER=\frac{1}{N}\overset{N}{\underset{k=1}{\sum }}\left(\frac{1}{6}\overset{6}{\underset{c=1}{\sum }}\left|\varepsilon_{c,k}\right|\right)$$

where “ε” is the strain; the subscript “c” identifies the strain components; the subscript “k” identifies the nodes of the DVC grid where the measurement is performed; N is the number of nodes of the DVC grid.

In order to consider the potential effect of the high heterogeneity of the local bone microstructure, a local analysis was performed within each vertebra on 27 sub-regions of interest defined within the VOI_DVC. Each vertebral body was divided in three longitudinal (xy-plane) regions of interest using a custom-made MATLAB script: top (most cranial ROIs), middle and bottom (most caudal ROIs). Then, each of them was divided into 9 sub-regions of interest (subROIs): anterior left (AL), anterior (A), anterior right (AR), left (L), central (C), right (R), posterior left (PL), posterior (P), posterior right (PR). The microstructural analyses were performed on each sub-region of interest (subROI). Each component of strain and the SDER were calculated within each subROI. SubROIs with volume lower than 1% of the total volume of the vertebral body were excluded from the sub-regional analysis.

In order to evaluate the errors and directionality for each specimen, we evaluated:

• Random error for each direction of displacement,
• Systematic error (mean) for each component of strain,
• Random error (standard deviation) for each component of strain,
• MAER (Mean Absolute Error),
• SDER (Standard Deviation of the Error).

In order to evaluate if a correlation exists between the microstructure of the bone and the performance of the DVC the following linear correlations were computed:

• The relationship between the displacement precision and four measurement spatial resolutions (NS of 25, 50, 75, or 100 voxels, equal to 0.97, 1.95, 2.92, and 3.90 mm, respectively) for metastatic and control vertebrae,
• The relationship between the SDER and four measurement spatial resolutions (NS of 25, 50, 75, or 100 voxels, equal to 0.97, 1.95, 2.92, and 3.90 mm, respectively) for metastatic and control vertebrae,
• The correlation between SDER for NS = 50 voxels and the microstructural parameters,
• The correlation between SDER for NS = 50 voxels and the microstructural parameters calculated in subROIs from metastatic and control vertebrae.

All the statistical analyses were performed using Prism (Prism 9, GraphPad Software, United States). Directionality of the displacement uncertainty was evaluated comparing the random errors among the different components of the displacement with a Kruskal-Wallis test. Directionality of the strain uncertainty was evaluated comparing the random error among the different components of strain with a Kruskal-Wallis test. Differences in the microstructural parameters between the entire dataset of metastatic vertebrae and control vertebrae dataset were tested with a Mann Whitney test. All statistical tests were performed with a level of significance equal to 0.05.

## 3 Results

### 3.1 Microstructural parameters

BV/TV, St.Th. And St.N. were significantly different in metastatic and control vertebrae (*p* < 0.01). St.Sp. Was not significantly different in metastatic and control vertebrae (*p* = 0.065).

### 3.2 Random errors for the displacements

**FIGURE 3:**
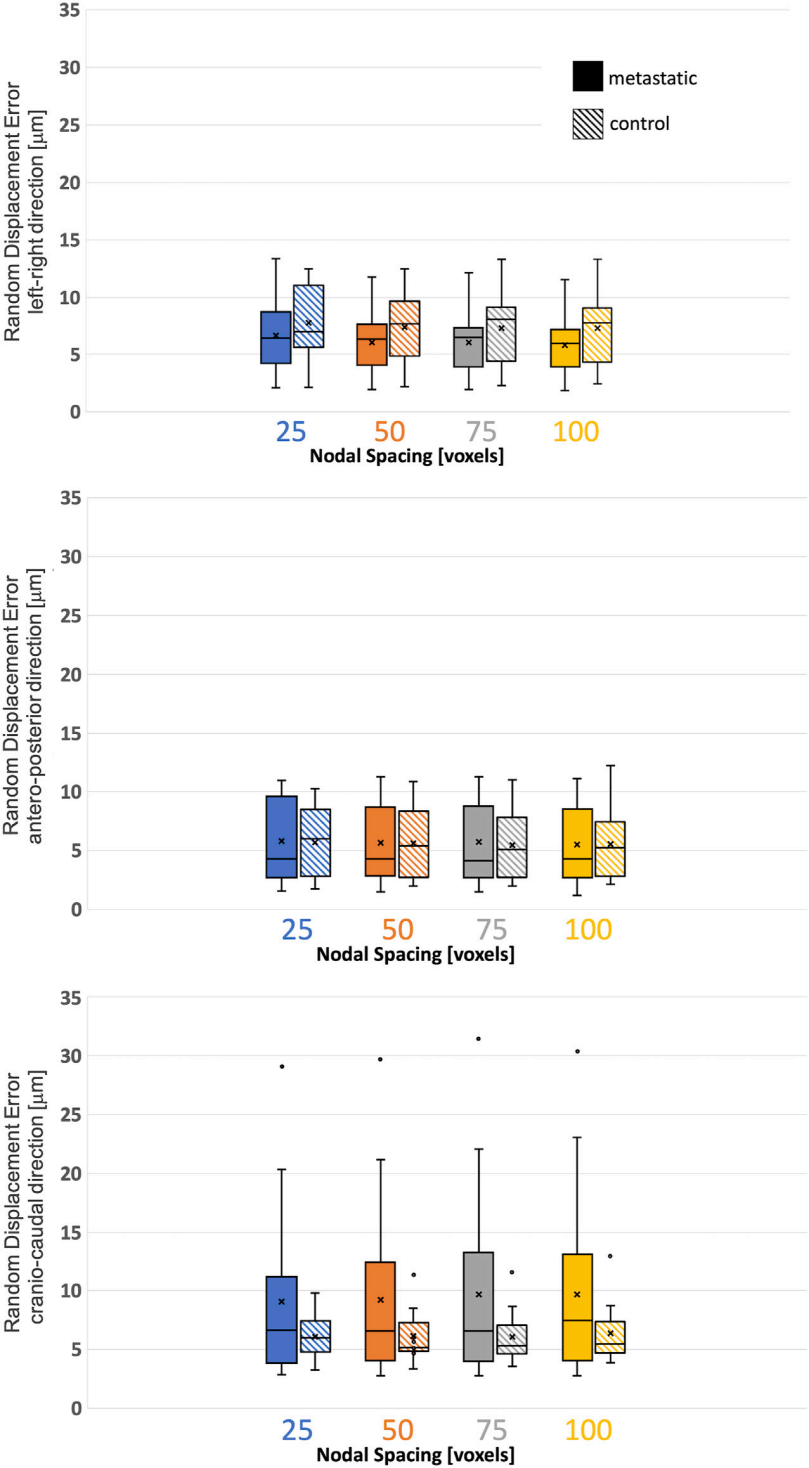
Variability of the displacement precision in metastatic and control vertebrae in VOI_DVC for different nodal spacing of 25 voxels (0.975 mm) in blue, 50 voxels (1.95 mm) in orange, 75 voxels (2.925 mm) in grey, and 100 voxels (3.9 mm) in yellow. The box is limited by the first and the third quartile. Whiskers represent the lowest and highest data point in the data set excluding any outliers (dots). Mean and median values among the group are represented by the cross and the horizontal line, respectively.

The random errors for each Cartesian component of displacement for each specimen are reported in detail in the Supplementary Materials. Isotropic distribution of the random errors was observed for all NSs (Kruskal-Wallis test, *p* > 0.05). The random error for each Cartesian component of the displacement and for the different NSs calculated in metastatic and control vertebrae were not significantly different (*p* > 0.2) (Figure 3; Table 3).

**TABLE 3:** Displacement random errors [mean ± standard deviation] reported in μm for the three Cartesian components of displacement for the different used NSs, for metastatic and control vertebrae.

Displacement random error [μm]	Displacement component	NS 25 voxels (0.975 mm)	NS 50voxels (1.95 mm)	NS 75 voxels (2.925 mm)	NS 100 voxels (3.9 mm)
Metastatic vertebrae	left-right	7 ± 3	6 ± 3	6 ± 3	6 ± 3
	antero-posterior	6 ± 3	6 ± 3	6 ± 3	5 ± 3
	cranio-caudal	9 ± 7	9 ± 8	10 ± 8	10 ± 8
Control vertebrae	left-right	8 ± 3	7 ± 3	7 ± 3	7 ± 3
	antero-posterior	6 ± 3	6 ± 3	5 ± 3	6 ± 3
	cranio-caudal	6 ± 2	6 ± 2	6 ± 2	6 ± 3

### 3.3 Standard deviation of the error (SDER)

**FIGURE 4:**
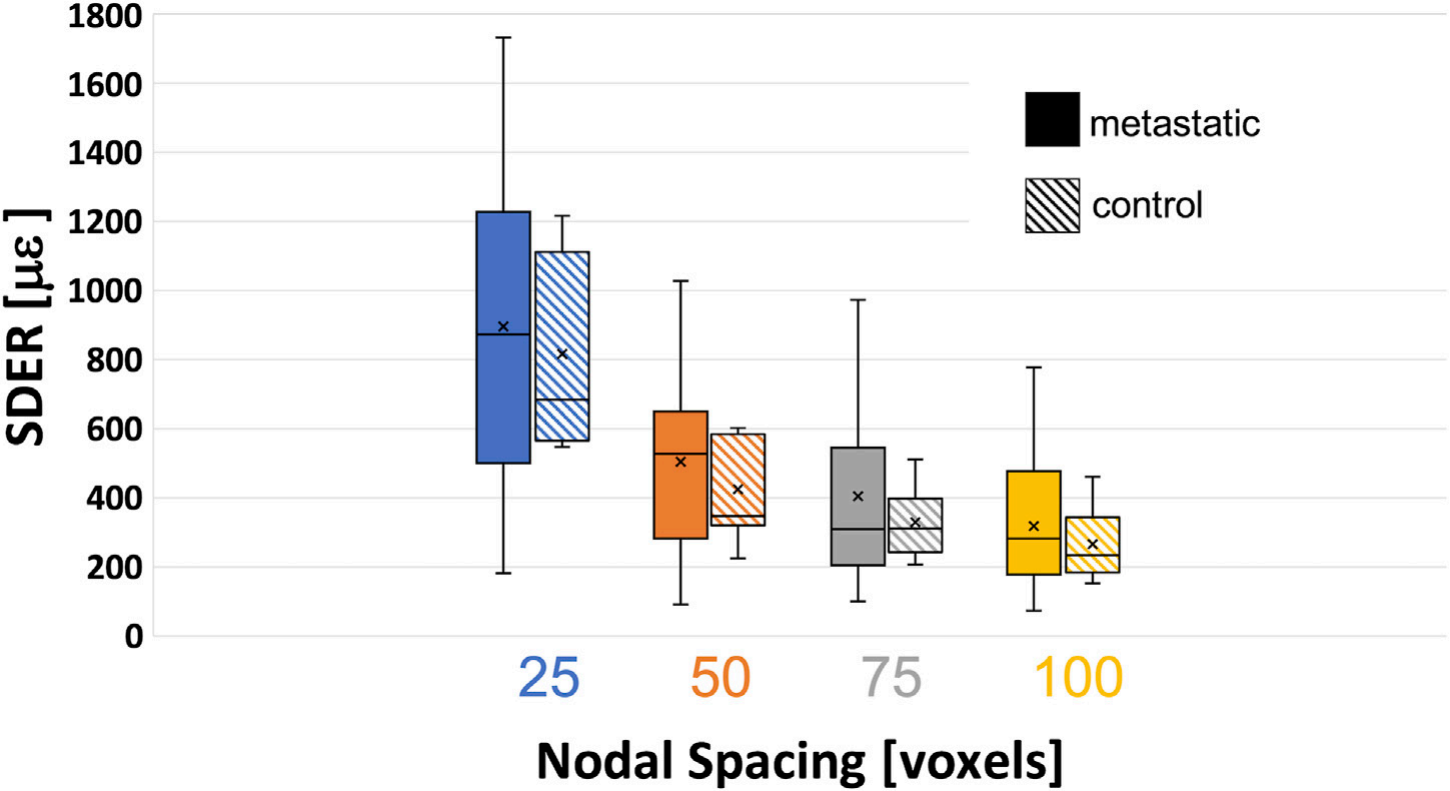
Variability of the standard deviation of the error (SDER) in metastatic and control vertebrae in VOI_DVC for different nodal spacing of 25 voxels (0.975 mm) in blue, 50 voxels (1.95 mm) in orange, 75 voxels (2.925 mm) in grey, and 100 voxels (3.9 mm) in yellow. The box is limited by the first and the third quartile. Whiskers represent the lowest and highest data point in the data set excluding any outliers. Mean and median values among the group are represented by the cross and the horizontal line, respectively.

The systematic and random errors for each Cartesian component of strain for each specimen are reported in detail in the Supplementary Materials. Anisotropic distribution of the random errors was observed for all NSs (Kruskal-Wallis test, *p* < 0.0001). In particular, lower errors were found along the antero-posterior and left-right directions (Supplementary Materials). SDER for the different NSs in metastatic and control vertebrae were not significantly different (*p* > 0.5) (Figure 4). The range of SDER in metastatic vertebrae were 182–1732 με, 91–1,030 με, 101–974 με and 75–777 με, for NS of 25, 50, 75, 100, respectively. In control vertebrae, the range of SDER were 548–1,213 με, 222–599 με, 205–512 με and 152–459 με, for NS of 25, 50, 75, and 100 voxels, respectively. The SDER showed a decreasing trend for larger NS.

### 3.4 Relationship between the local microstructural parameters and the SDER

**FIGURE 5:**
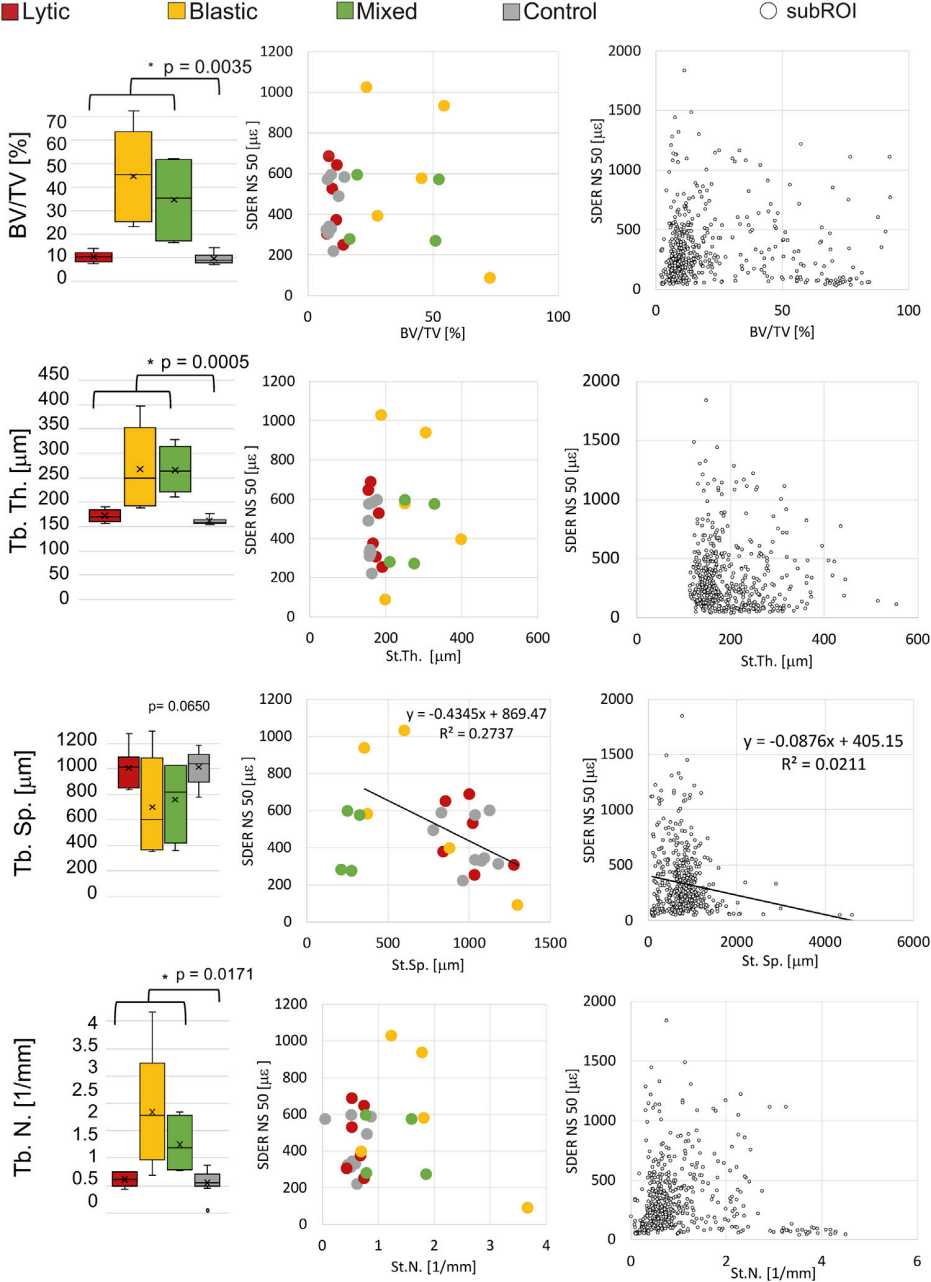
Left column: Boxplot of the microstructural parameters of metastatic vertebrae, grouped in vertebrae with lytic (red), blastic (yellow) and mixed (green) metastases, and control (grey) vertebrae. The box is limited by the first and the third quartile. Whiskers represent the lowest and highest data point in the data set excluding any outliers (dots). Mean and median values among the group are represented by the cross and the horizontal line, respectively. Statistically significant differences (Mann-Whitney test) between the whole metastatic group and control group are highlighted with *. Central column: relationship between the microstructural parameters and the SDER evaluated on the whole vertebra, vertebrae with lytic (red), blastic (yellow) and mixed (green) metastases and control (grey) vertebrae. Right column: relationship between the microstructural parameters and the SDER evaluated in the local subROIs (data pooled for metastatic and control vertebrae).

NS of 50 voxels was chosen as the smallest measurement spatial resolution that produced acceptable errors for measuring strain at failure (Dall’Ara et al., 2014; Palanca et al., 2017). The correlation between the SDER (in the VOI_DVC, NS = 50 voxels) and the microstructural parameters (in the VOI_NoCort) were not statistically significant except for a weak correlation between the SDER and the St.Sp. In metastatic vertebrae (*p*-values = 0.045, *R* ^2^ = 0.27, Slope = 0.43, Intercept = 869) (Figure 5). For the sub-regional analysis 119 subROIs out of 648 were excluded as they had a volume lower than 1% of the whole vertebral body. No significant correlation was found between the microstructural parameters and the SDER (NS = 50, remaining 529 subROIs, *p* > 0.4), with exception of a very weak correlation between the SDER and the St.Sp. (*p*-value = 0.0008, *R* ^2^ = 0.02, Slope = −0.09, Intercept = 405) (Figure 5).

**FIGURE 6:**
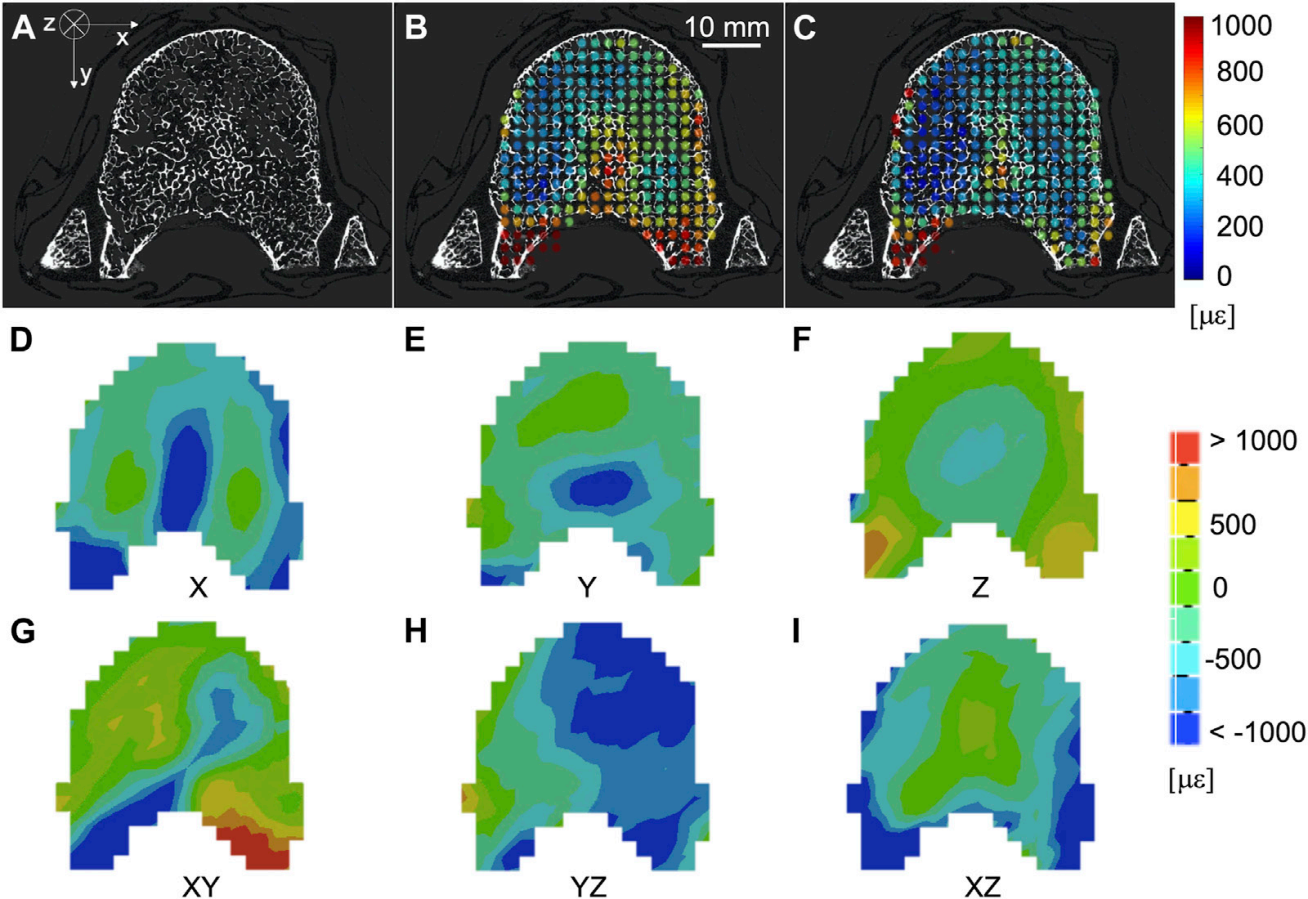
**(A)** microCT cross section of a healthy vertebra. Spatial distribution of the mean **(B)** and standard deviation **(C)** of the absolute value of the six components of the strain [με] (NS = 50 voxels) on the same microCT cross section. From **(D–I)** spatial distribution of the six components of the strain [με] (NS = 50 voxels) on the same microCT cross section.

**FIGURE 7:**
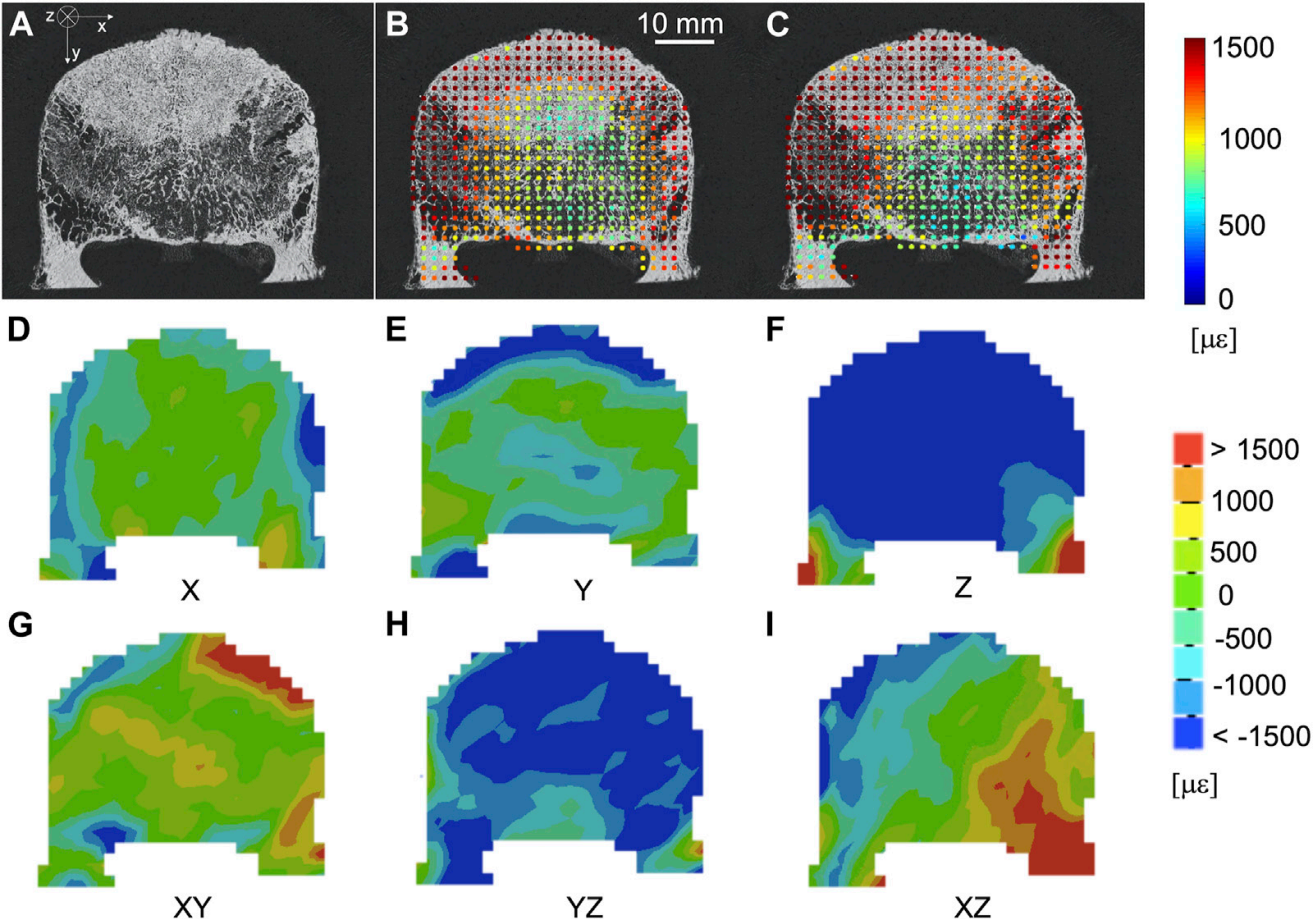
**(A)** microCT cross section of a vertebra with blastic metastases (indicated by a white arrow) in the anterior portion of the vertebral body. Spatial distribution of the mean **(B)** and standard deviation **(C)** of the absolute value of the six components of strain [με] (NS = 50 voxels) on the same microCT cross section. From **(D–I)** spatial distribution of the six components of the strain [με] (NS = 50 voxels) on the same microCT cross section.

The spatial distribution of the mean and standard deviation of the absolute value of the six components of the strain for NS = 50 voxels was evaluated for healthy (Figures 6A–C) and metastatic (Figures 7A–C) vertebrae. The distribution of the measurement uncertainties for each Cartesian component of the strain was evaluated through 3D strain colour maps (Mechanical APDL v19, ANSYS, United States) for healthy (Figures 6D–I) and metastatic (Figures 7D–I) vertebrae.

## 4 Discussion

The aim of the study was to investigate if the microstructural properties of the trabecular microstructure can affect the measurement uncertainties of a global DVC approach [BoneDVC (Dall’Ara et al., 2014)]. In particular, vertebrae with and without metastases were used to provide microstructural variability and four different three-dimensional microstructural parameters (i.e., BV/TV, St.Th., St.Sp. And St.N.) were investigated.

Even if significant differences in the microstructure of metastatic and control vertebrae were found, the displacement and strain measurement uncertainties were not significantly different in metastatic and control vertebrae. In this study, the displacement uncertainty was isotropic and with a magnitude always below the voxel size (i.e. 39 μm). The displacement uncertainties in metastatic vertebrae were higher (range: 1−30 μm) than the uncertainties in control vertebrae (range: 2–12 μm). On this regard, four metastatic vertebrae and one control vertebra were associated with random displacement errors larger than 10 μm but no clear microstructural features or specific event explained this behaviour. Random errors of the strain showed an anisotropic behaviour, smaller errors were observed in the anterior-posterior and left-right directions, as reported in (Palanca et al., 2015). The SDER ranged 91–1030 με and 222–599 με in metastatic and control vertebrae, respectively. As hypothesized in (Dall’Ara et al., 2014) global DVC approaches (in this case BoneDVC) are minimally affected by the local microstructures, as long as there is enough heterogeneity in the structure. However, this low sensitivity to microstructural properties is partially in contrast with the results obtained by (Liu and Morgan, 2007), who highlighted the importance of considering the specimen density and trabecular microstructure of the type of bone when using a local DVC approach. In particular, they showed lower measurement uncertainties associated with lower BV/TV, Trabecular Thickness and Trabecular Number. Nevertheless, in that study different bone types from different species and anatomical sites were investigated, which may explain the different findings.

**FIGURE 8:**
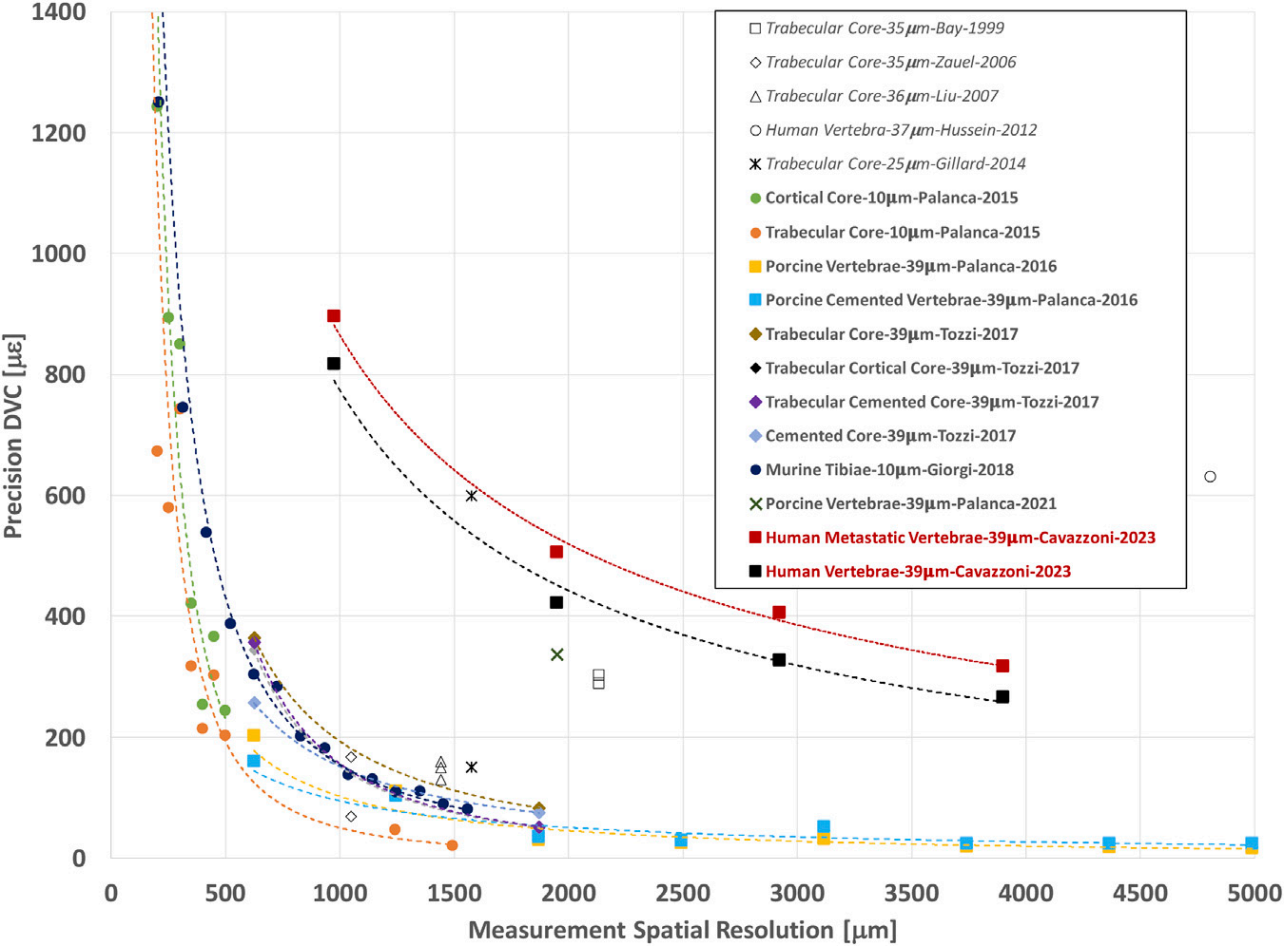
Relationship between the precision (SDER; median values in case more specimens were analysed per group) and the spatial resolution of the DVC strain measurement (in μm). The spatial resolution of the DVC is equivalent to the NS used in this study. In other studies, it is referred to Sub-Volume size or similar. For each study the specimen type, microCT image resolution, first author of the manuscript, and year of publication of the manuscript are reported. Study performed with BoneDVC approach are reported in bold and study performed with other approaches are reported in italics.

In this study, both displacement and strain measurement uncertainties tend to decrease when NS increases. Several studies (Figure 8) showed similar trends for different types of bones, like cortical and trabecular bone cores, and porcine vertebrae (Bay et al., 1999; Zauel et al., 2006; Dall’Ara et al., 2014; Gillard et al., 2014; Palanca et al., 2015; Palanca et al., 2016) for similar range of NS. Indeed, the chosen measurement spatial resolution has to be large enough to include a volume with a univocal trabecular pattern and thus a univocal grey scale gradient intensity in order to distinguish this region from the others (Dall’Ara et al., 2014; Palanca et al., 2015). At the same time, the measurement spatial resolution should be small enough to discriminate gradients in displacement and strain field within the analysed bone. A nodal spacing of 50 voxels was identified as the best compromise between the SDER and the measurement spatial resolution as it is the smallest measurement spatial resolution that ensure acceptably low measurement uncertainties, at least an order of magnitude lower than those at failure (∼7000 με in tensile and ∼10000 με in compression (Morgan and Keaveny, 2001)). These results were comparable to those found in previous studies (Figure 8) on porcine vertebrae imaged with the same microCT scanner of this study at 39 μm with spatial resolution of 50 voxels (1.95 mm) for which SDER was found equal to 337 με (Palanca et al., 2021b), and whole human vertebrae scanned at 37 μm with sub-volumes side length ∼4.8 mm with measurement uncertainties of 630 με (Hussein et al., 2012). Nevertheless, the SDER found in this study was worse than those found in other DVC studies that evaluated the strain measurement uncertainty on whole vertebrae (Palanca et al., 2015) and bone cores (Gillard et al., 2014; Palanca et al., 2015; Tozzi et al., 2017) (Figure 8), using industrial microCT scanners. The difference may be due to the higher power used in those studies to scan the bone, which is not achievable with the *in vivo* microCT scanner used in this study.

The correlation analysis showed that the SDER (NS = 50 voxels) was only weakly correlated with the St.Sp. In metastatic vertebrae: vertebrae characterized by a denser trabecular microstructure, typical of blastic lesions, were associated with higher values of SDER; vertebrae with lower density (low BV/TV and high St.Sp.), typical of vertebrae with lytic lesions, were associated with lower values of SDER. This result could appear in conflict with those reported in other studies using a similar DVC approach on bone core specimens scanned at higher resolution (Dall’Ara et al., 2014; Palanca et al., 2015). In fact, they showed that cortical bone specimens, which are characterized by a denser and more homogeneous bone, similar to blastic lesions, had a better precision (NS = 50 voxels) than less dense trabecular bone tissue. This could be explained by the different spatial resolution of the scans (9.96 μm vs. 39 μm), that allowed to resolve more microstructural features in the cortical bone, and thus grayscales with higher gradients, that helped the image registration.

Since average microstructural parameters did not represent the variability of the microstructure, and did not explain the variability of the measurement uncertainty, a local qualitative investigation of the spatial distribution of the SDER against microstructure was performed. In particular, larger SDER values were observed in regions lacking microstructural features such as outer boundaries and regions within blastic lesions. In these regions, the resolution of the microCT scans (39 μm) could not resolve microstructural features creating low gradients of greyscale values (Figures 6, 7). Similar distributions were observed by (Palanca et al., 2016) on whole porcine vertebrae: measurement uncertainties tended to increase at the outer boundaries of the bone, where the thick cortical shell was located. On the contrary, in case of regions including high greyscale gradients lower SDER values were observed, highlighting the association between the spatial distribution of the microstructure and the spatial distribution of the SDER. A more homogeneous spatial distribution of the SDER, indeed, corresponded to the more homogeneous microstructure of the control vertebrae.

Considering the great variability and heterogeneity of the microstructure within metastatic vertebrae a local analysis was performed on subROIs. This analysis confirms the weak correlation between the SDER (NS = 50 voxels) and the St.Sp., (larger the St.Sp., lower the SDER) highlighting the low sensitivity of the global DVC approach, to the analysed microstructural properties of the tissue scanned at this resolution.

The ranges of measurement uncertainties obtained for both metastatic and control vertebrae enable the application of the DVC approach to study the strain field within metastatic vertebral bodies, as performed on healthy vertebrae (Hussein et al., 2012), in particular to discriminate those regions which experience failure and those which not (Morgan and Keaveny, 2001). Nevertheless, as remarked in other studies and confirmed in this work, these measurement uncertainties may be quite different from specimen to specimen with heterogeneous structures and need to be assessed in each specimen, at least with zero-strain test from repeated scans, before running DVC analyses of the specimen under different load levels.

This study has some limitations. The measurement uncertainties were estimated only with the specimen in the unloaded condition, without considering how the uncertainties would be affected by different loads. While virtual simplified (affine) deformations on repeated scans have been proposed in DVC uncertainties studies (Comini et al., 2019; Ryan et al., 2020), they just highlighted artificial high errors at the boundary of the image. Despite a large number of heterogeneous specimens was analysed, due to the relatively small group size for different types of lesions, it was not possible to evaluate potential differences in uncertainties between vertebral body with lytic, blastic or mixed lesions. Only few microstructural parameters were investigated: further analyses should include other parameters such as the Degree of Anisotropy (DA), Connectivity Density (Conn. D), the Bone Surface (BS), the relative Bone Surface (BS/TV) and the Structural Model Index (SMI) (Ulrich et al., 1999; Liu and Morgan, 2007). Moreover, specimens were selected so that the metastases did not involve the cortical shell because in this case it would not have been possible to define the boundary of the VOIs. Due to the size of the specimens it was not possible to increase the resolution of the microCT images and scan the whole vertebral body in a reasonable time. Nevertheless, the microCT-acquisition protocol applied in this study enabled the evaluation of the 3D displacement and strain fields on whole human vertebral bodies, with acceptable precision and spatial resolution. Finally, the authors are aware that other sources of errors exist: such as the quality of the input images (image resolution, artefacts, SNR) and the correlation algorithm (objective and shape function, operational parameters) (Roberts et al., 2014). The effect of them have already been partially investigated through a zero-strain analysis, in different bone structures (Dall’Ara and Tozzi, 2022).

In conclusion, bone microstructure in both healthy and metastatic human vertebral bodies has only a weak effect on the measurement uncertainties of a global DVC algorithm. Nevertheless, the authors suggest to perform a preliminary analysis of the measurement uncertainty, e.g., in zero-strain condition, for each specimen before evaluating the strain under load. This analysis will be helpful identify the minimum unavoidable error associated with the DVC measurements and better interpret the DVC results.

## Data Availability

The original contributions presented in the study are included in the article/Supplementary Material, further inquiries can be directed to the corresponding author.

## Fundings

The study was partially supported by the AOSpine Discovery and Innovation Awards (AOSDIA 2019_063_TUM_MP), Marie Skłodowska-Curie Individual Fellowship (MetaSpine, MSCA-IF-EF-ST, 832430/2018) and by the Engineering and Physical Sciences Research Council (EPSRC) Frontier Multisim Grant (EP/K03877X/1 and EP/S032940/1).
